# Development of a phenotyping protocol for combined drought and salinity stress at seedling stage in rice

**DOI:** 10.3389/fpls.2023.1173012

**Published:** 2023-05-31

**Authors:** Suneetha Kota, Naireen Aiza Vispo, Marinell R. Quintana, Carlo L. U. Cabral, C. Arloo Centeno, James Egdane, Frans J. M. Maathuis, Ajay Kohli, Amelia Henry, Rakesh Kumar Singh

**Affiliations:** ^1^ Rice Breeding Innovations Department, International Rice Research Institute, Los Baños, Laguna, Philippines; ^2^ Plant Breeding, Indian Institute of Rice Research, Hyderabad, Telangana, India; ^3^ Biology Department, University of York, York, United Kingdom

**Keywords:** rice, combined stress, drought, salinity, chlorophyll fluorescence, abiotic stress

## Abstract

**Introduction:**

The case of combined drought and salinity stress is increasingly becoming a constraint to rice production, especially in coastal areas and river deltas where low rainfall not only reduces soil moisture levels but also reduces the flow of river water, resulting in intrusion of saline sea-water. A standardized screening method is needed in order to systematically evaluate rice cultivars under combined drought+salinity at the same time because sequential stress of salinity followed by drought or vice-versa is not similar to simultaneous stress effects. Therefore, we aimed to develop a screening protocol for combined drought+salinity stress applied to soil-grown plants at seedling stage.

**Methods:**

The study system used 30-L soil-filled boxes, which allowed a comparison of plant growth under control conditions, individual drought and salinity stress, as well as combined drought+salinity. A set of salinity tolerant and drought tolerant cultivars were tested, together with several popular but salinity and drought-susceptible varieties that are grown in regions prone to combined drought+salinity. A range of treatments were tested including different timings of the drought and salinity application, and different severities of stress, in order to determine the most effective that resulted in visible distinction among cultivars. The challenges related to determining a protocol with repeatable seedling stage stress treatment effects while achieving a uniform plant stand are described here.

**Results:**

The optimized protocol simultaneously applied both stresses by planting into saline soil at 75% of field capacity which was then allowed to undergo progressive drydown. Meanwhile, physiological characterization revealed that chlorophyll fluorescence at seedling stage correlated well with grain yield when drought stress was applied to vegetative stage only.

**Discussion:**

The drought+salinity protocol developed here can be used for screening rice breeding populations as part of a pipeline to develop new rice varieties with improved adaptation to combined stresses.

## 1 Introduction

Abiotic stresses limit rice crop production worldwide (Shahbaz and Ashraf, 2013; Almeida et al., 2016). Simultaneous exposure to multiple abiotic stresses at a given time, for example drought and salinity, salinity and submergence, or salinity-submergence-drought is becoming more frequent (Takeda and Matsuoka, 2008). The case of combined drought and salinity stress is particularly observed in coastal areas. For example, low rainfall resulting in drought in the Mekong River Delta of Vietnam results in intrusion of saline sea-water to areas used for crop cultivation (Larson, 2016), with about 1.8 million ha of the growing area subjected to increased dry-season salinity (Smajgl et al., 2015). To address yield losses, progress on breeding for tolerance to individual abiotic stresses such as drought, salinity, and flooding has been significant, and several tolerant varieties suitable for specific stresses have been developed and made available for farmer cultivation (Gregorio et al., 2013; Amaranatha et al., 2014; Hoque et al., 2015; Singh et al., 2016; Waziri et al., 2016; Dar et al., 2018). However, little progress on development of varieties tolerant to combined abiotic stresses has been reported.

Drought and salinity are two major abiotic stresses that show some degree of similar physiological effects on the rice plant (Furbank and Tester, 2011). When seedlings are exposed to salinity, the induced osmotic stress results in partial closure of stomata that reduces water uptake by the roots from the soil within hours to days of exposure (Munns et al., 2010). Similarly, plants experience the osmotic effects of drought stress when evapotranspiration demand exceeds soil moisture availability (Pinheiro and Chaves, 2011; Ayalew et al., 2014; Basu et al., 2017; Farquharson, 2017; Zhang et al., 2018). Breeding for tolerance to both drought and salinity, with their intricate and multifaceted overlapping mechanisms, requires a standardized protocol to impose both stresses at the same time to screen and understand the plant responses.

Phenotyping is the most critical component in identification of potential donors for varietal development through genetic improvement, and a range of protocols and targeted growth stages have been used for both drought and salinity screening. Withholding soil moisture in field or pot culture and the addition of polyethylene glycol (PEG) to the growth medium are the most widely adopted methods (Michel and Kaufmann, 1973). Typically, solution and soil culture methods have been used for early growth stage screening, and pot and field experiments have been used for late growth stage screening (Kranto et al., 2016). The Yoshida solution culture method described by Gregorio et al. (1997) has been extensively used as a rapid method for screening large numbers of genotypes at the seedling stage. The method typically employs perforated styrofoam sealed underneath with a net, in which seedlings are planted and floated on the solution (Singh et al., 2010). Salt in the form of NaCl (to impose salinity stress) or PEG (as a general osmotic stress agent for both drought and salinity screening) are added to the solution (Guan et al., 2010; Lima et al., 2015; Basu et al., 2017). Plants are subsequently evaluated to differentiate the tolerant from the sensitive genotypes.

In salinity treatments, visual symptoms of salt stress are typically evident whereas in drought treatments, visual evaluation in seedling stage screens is not straightforward and can show interactions with plant biomass (Mitchell et al., 1998). Therefore, physiological measurements may be a more reliable screening tool as compared to visual scores. For example, chlorophyll fluorescence has been used as a surrogate measurement for maintenance of photosynthetic function under stresses such as drought (Baker, 2008; Hasanuzzaman et al., 2019), salinity (Tsai et al., 2019), and even recently for combined stresses of salinity and submergence (Pradhan et al., 2019).

Given the importance of combined drought and salinity in farmers’ fields in coastal rice-growing regions, as well as the lack of standardized protocols for evaluating rice under combined stresses, in this study we aimed to develop a method to screen rice genotypes under the combination of drought and salinity stress. Although abiotic stress may occur at any growth stage of the rice crop, our objective was to screen the rice genotypes at seedling stage for stress survival as well as to conduct physiological characterization as a pilot study that could subsequently be scaled up to later growth stages. By characterizing a set of cultivars with known tolerance to individual abiotic stresses, we explored which salinity tolerant and drought tolerant cultivars would be most effective in improving rice tolerance to combined drought+salinity stress.

## 2 Materials and methods

In this study, we developed a screening method for combined tolerance to drought and salinity stresses while characterizing the physiological stress response under individual drought and salinity stresses as well as under the optimized protocol for drought+salinity stress.

### 2.1 Plant materials

This research was conducted using eight rice cultivars: FL 478 (IR 66946-3R-178-1-1) and CSR 28 as salinity tolerant lines, Sukha dhan 6 (IR83383-B-B-129-4) and Sahbhagi dhan (IR74371-70-1-1) as drought tolerant lines (Dar et al., 2014), IRRI 154 (NSIC Rc222) and IRRI 141 (Anjelica) as high yielding popular varieties in the Philippines, OM 4900 as a popular high yielding Vietnamese variety, and IR 29 as the salinity sensitive check. This set was selected to include drought tolerant and salinity tolerant cultivars as well as popular (but salt and drought susceptible) varieties that are grown in regions where combined drought+salinity may occur.

### 2.2 Development of a standardized protocol to impose combined drought+salinity stress

**Figure 1:**
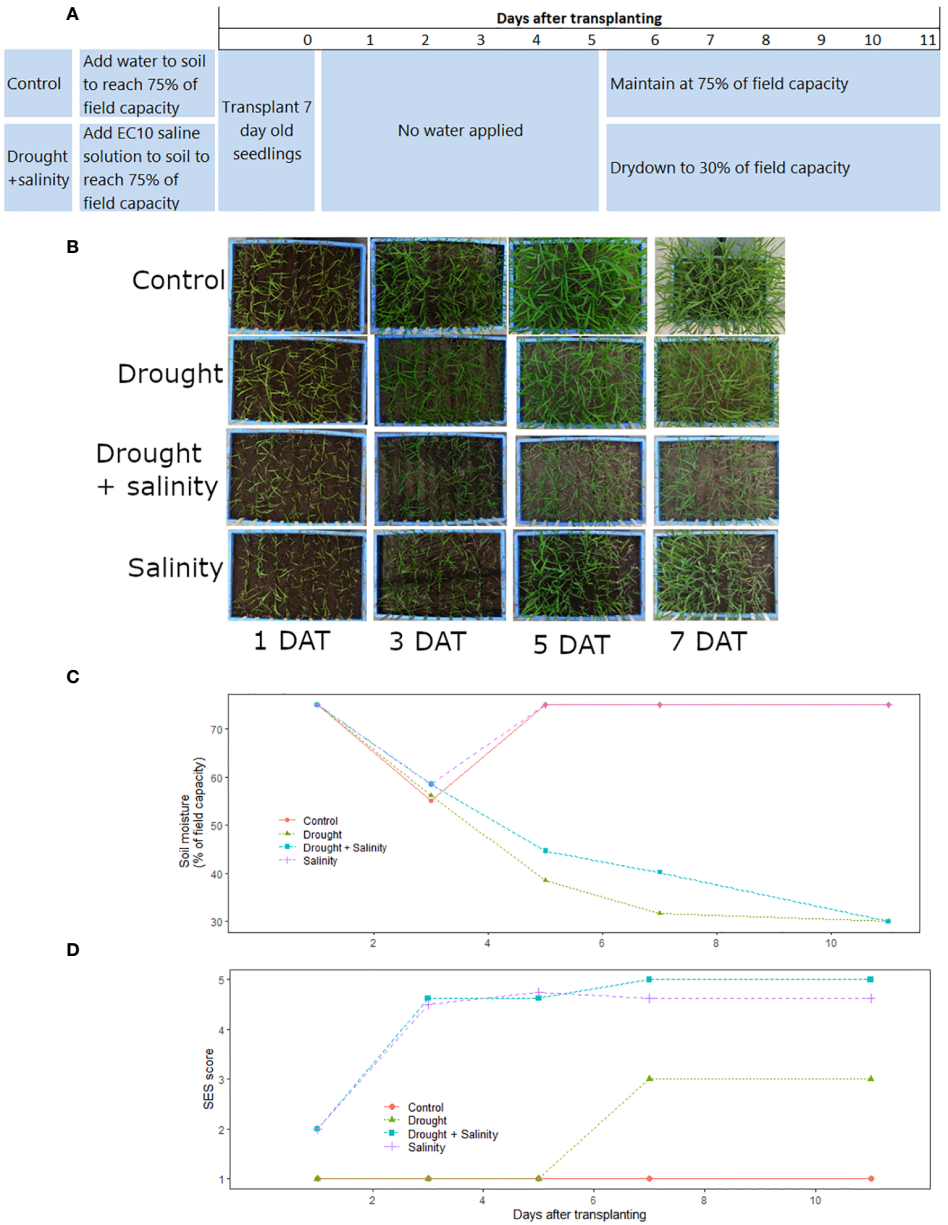
Timeline of the optimized protocol for combined drought+salinity stress screening in terms of **(A)** planting and treatment applications in the drought+salinity and control treatments, **(B)** overhead images of the treatment progressions over time, **(C)** soil moisture over time in each treatment, **(D)** SES scores (all eight cultivars averaged) over time in each treatment. The salinity (T29), drought (T30), drought + salinity (T31), and control (T32) treatments shown here are described in **Supplementary Table 1**). Data and images shown are from the Phytotron January 2019 experiment.

Optimization experiments to develop a phenotyping protocol for combined drought+salinity stress (together with individual drought and salinity treatments for comparison) were conducted using soil-filled trays. Our aim was to impose stress on the test cultivars grown together in the same pool of soil to minimize variation in soil moisture levels experienced by each cultivar, and to target seedling stage in order to avoid interactions with varying phenology among cultivars. Repeated experiments were conducted with treatments of drought stress alone, salinity stress alone, drought first followed by salinity stress, salinity first followed by drought stress and a combination of both stress together tested (**Supplementary Table 1**), with more than 30 treatments evaluated over the course of eight separate experiments from July 2017-January 2019. The protocol resulting in good seedling establishment while allowing visually observable progression of stress symptoms and distinct responses among the four treatments (control, drought alone, salinity alone, and drought+salinity) was selected and is described below and in **Figure 1**. All eight selected cultivars were used in the optimization of the standardized drought+salinity protocol.

For the optimized drought+salinity protocol, the experiment was set up in trays of 30 L (56.5 cm x 36.5 cm x 15 cm) filled with sieved, sterilized soil at a bulk density of 1.0 g cm^-3^. The amount of water to achieve field capacity of the soil was determined before the start of experiment. Seeds were surface sterilized by wrapping them in paper towel, soaking in 5% sodium hypochlorite solution for 5-10 minutes, and then rinsing thrice with water. The seeds were then wrapped in another paper towel and soaked in water for 72 hours for germination. The germinated seeds were sown into seedling trays (10 L volume, 33.5 cm x 26.3 cm x 11.0 cm) filled with sterilized soil. After emergence, seedlings at the 2-3 leaf stage were transplanted in nine rows (one row per cultivar, except for IR29 which was planted in one row on each end of the tray) into the experimental trays with 7 cm spacing between rows and 20 seedlings per row. The initial soil moisture level was at 75% of field capacity (FC) in all treatments. In the control and drought treatments, 75% of FC was reached by adding tap water to the dry soil, and in the salinity and drought+salinity treatments saline solution (the amount of salt required to reach an electrical conductivity (EC) of 10 dS m^-1^ at 100% of FC dissolved in the amount of water required for 75% of FC) was added to the dry soil before planting. The drought and drought+salinity stress treatments were allowed to dry down until the end of the experiment or until the soil moisture level reached 30% of FC, at which time tap water was added to maintain the soil moisture at 30% of FC. The soil moisture levels were monitored by weighing (Kern FKB 65k1A) every other day to maintain the reference weight corresponding to the targeted soil water status treatment. To reduce cracking of the soil that could occur while moving the experimental trays, wooden planks fitted with handles were kept under each tray during the course of the study that were used to carry each tray to the balance. The experiments were carried out in controlled environmental conditions of 34/25°C day/night temperature with a relative humidity of 70% in the Phytotron facility at the International Rice Research Institute (IRRI; Los Baños, Laguna, Philippines (14°10′N, 121°15′E)).

To characterize the plant responses to each treatment in the standardized protocol, visual scores based on the Standard evaluation system (SES; IRRI, 1996) were recorded on 3, 5, 7, 11 and 12 days after transplanting to monitor the visual symptoms of stress of salinity and drought. After termination of the experiment, soil samples from the top, middle and bottom layers of the experimental trays were collected and the soil EC levels were determined from a 1:2 soil: distilled water ratio. The resulting soil extract EC (ECe) was calculated by multiplying the EC meter reading in dSm^-1^ by 5 according to Aboukila and Abdelaty (2017).

### 2.3 Physiological characterization of drought and salinity tolerant cultivars and popular varieties

To characterize the stress response of the cultivars in this study and to evaluate proxy measures for stress tolerance that could be used for seedling stage stress screening, physiological characterization was conducted in both an experimental tray system in a Phytotron (while the drought+salinity protocol was being optimized) and in a field trial. The physiological characterization in Phytotron experiments was conducted in October, 2017 under treatments T5, T12, T13 and T16, in November, 2017 under treatments T3, T14, T15 and T16, in July 2018 under treatments T29, T30, and T31, and in January 2019 under treatments T29, T30, T31, and T32 (as described below and in **Supplementary Table 1**). The field trial was grown in the 2018 dry season (Jan – April) in an open field at the IRRI Zeigler Experiment Station.

In addition to a seedling stage drought treatment, the field physiology trial also included a rainfed treatment, which was allowed to dry down throughout the season and was only rewatered on 46 and 88 days after sowing (DAS), and a well-watered control treatment that was maintained flooded throughout the season. The seedling stage drought stress field trial was re-watered at 45 DAS and maintained well-watered throughout the season thereafter. Each of the eight rice cultivars were transplanted in the field in three 3-m row plots with a spacing of 20 cm between hills and 25 cm between rows, and four replicates per cultivar in a randomized complete block design. At the time of seedling stage measurements, the soil moisture levels were close to 30% of field capacity in the Phytotron experiments (**Supplementary Figure 1**) and averaged 14% gravimetric moisture content in the field (soil depth: 0-15 cm). Seedling/early vegetative stage shoot biomass, chlorophyll fluorescence (two light-adapted leaves per plant, Walz Mini PAM, with 20 s illumination before each measurement), and chlorophyll concentration index (Apogee Instruments Chlorophyll Content Meter) were measured in both the Phytotron (18 DAS) and field (45 DAS) experiments. Standard evaluation system (SES) scores were recorded in the Phytotron experiments at 18 DAS. Levels of Na^+^ and K^+^ were determined from the dried shoot tissue of three plants per cultivar in the Phytotron experiments; 10 mg of ground shoot tissue was placed in a vial with 10 ml of 100mM acetic acid, digested for two hours in a 90°C water bath, filtered, and the filtrate was diluted 10× (9mL nanopure water and 1mL filtrate) and ionic concentrations were measured using an atomic absorption spectrophotometer (PerkinElmer Analyst 300) as described by Calapit-Palao et al. (2013) and Ahmadizadeh et al. (2016). In the field trial, reproductive stage measurements of chlorophyll fluorescence, chlorophyll concentration index, stomatal conductance (2 leaves per plot; AP4, Delta-T, UK), and canopy temperature (3 locations per plot; Apogee Instruments Infrared sensor, Logan UT, USA) were conducted at 101 DAS. At crop maturity, straw biomass and grains from the field trial were harvested from a 1.5 m^2^ area of each plot and grain yield was calculated based on a 14% moisture content.

### 2.4 Statistical analysis

In the physiology experiments, the percent reduction as compared to the control was calculated for the traits measured in the stress treatments as: $\frac{x_{stress}-{\bar{x}}_{control}}{{\bar{x}}_{control}}\times 100$ . Cultivar means were compared by ANOVA followed by Tukey’s test, and correlations among traits were determined by Pearson correlation using the *agricolae* package in R v. 4.0.3 (R Core Team, 2017).

## 3 Results

### 3.1 Optimization of a standardized protocol to impose combined drought+salinity stress

Our protocol optimization strategy involved identification of the individual stress levels to be applied in the soil-filled tray experimental setup, followed by evaluation of the plant response to a range of combinations of drought and salinity at seedling stage (**Supplementary Table 1**). Several considerations were deduced across this series of treatments applied in the optimization process, particularly regarding the level of stress applied as affected by the soil moisture content, the method of seedling establishment (seeding of pre-germinated seeds/dry direct seeding/transplanting), and the relative humidity and temperature of the growth environment. For the combined drought+salinity stress treatment, the timing and order of stress imposition influenced the manifestation of stress symptoms and the ability to differentiate the tolerant and sensitive cultivars. Each of these factors was tested in the series of the optimization experiments.

Our series of optimization experiments was aimed at the following: A) testing different levels of individual salinity stress, B) testing different orders of applying salinity and drought stress, with different levels of salinity but all soil moisture levels starting at field capacity, C) testing treatments initiated at soil moisture levels below field capacity, D) testing treatments allowed to dry down to 30% of field capacity only, E) monitoring soil moisture evaporation in unplanted trays across different treatments, F) comparing direct seeding with transplanting young seedlings, and E) finalizing the optimized seedling stage drought+ salinity protocol (**Supplementary Table 1**).

For the individual salinity treatment, adding solution with an electrical conductivity of 10 dS m^-1^ (EC 10) allowed the best visual distinction between the known salinity-tolerant and -sensitive cultivars. The known tolerant and sensitive cultivars could not be distinguished in treatments using saline solution of EC 4 due to the relatively weak stress level. Phenotypically, the EC 8 dS m^-1^ stress level was sufficient to differentiate the cultivars into sensitive, moderately tolerant and tolerant categories, while EC 10 dSm^-1^ allowed classification into tolerant and sensitive categories, and adding solution of EC 12 dSm^-1^ resulted in a strong stress level in which all the cultivars except the tolerant checks started to wilt within a few days of stress imposition. For the individual drought treatment, we found that a progressive drydown treatment resulted in variable soil moisture levels across repetitions; we therefore targeted the minimum soil moisture level to be maintained at 30% of field capacity. We also observed that the drydown progressed slowly in some repetitions and therefore initiated the drought treatment at 75% of field capacity so that a treatment effect could be observed within the short duration (~12 days) of each trial.

Differences in seedling emergence and establishment were observed in treatments with pre-germinated seeds as well as dry direct seeding. Although direct seeding exhibited an advantage in the speed of germination, the establishment tended to be non-uniform and direct seeding into soil that was salinized with reduced soil moisture resulted in poor seedling emergence. We therefore shifted the protocol to transplanting 7 day old seedlings.

We also observed that the ambient temperature had a strong effect on the development of visible salinity stress symptoms; treatments conducted at 30 or 32°C showed few visual symptoms whereas the symptoms observed in treatments conducted at 34°C were much more distinct. The temperature regimes increasing to a maximum of 34°C in combination with a relative humidity of 70% was found to be optimum for stress imposition.

The optimization of the combined drought+salinity treatment proved to be most challenging due to the opposing nature of requiring dry soil for drought treatments and addition of saline solution for salinity treatments. We first aimed to apply one stress at a time, but this did not allow stress response symptoms (visual or physiological) to develop within our targeted timeframe. Interestingly, we observed a notable difference in the order of individual stress application; when salinity stress was applied first followed by drought, the level of stress became overly severe. When drought was applied followed by salinity, a longer duration was required and the older and larger seedlings were more prone to resisting the subsequent salinity stress. We therefore concluded that both stresses should be applied simultaneously by planting into saline soil at 75% of field capacity which was then allowed to undergo progressive drydown.

### 3.2 Physiological characterization of salinity and drought tolerant cultivars and popular varieties

The soil moisture level (**Figure 1B**; **Supplementary Figure 1**) and soil electrical conductivity (**Supplementary Table 4**) varied among treatments. The soil moisture levels declined slightly less in the drought+salinity treatments than in the treatments with drought alone (**Figure 1B**; **Supplementary Figure 1**). The EC of the soil was generally slightly higher in the drought+salinity treatments than in the treatments with salinity alone, and the EC level of the upper soil tended to be higher than that of the soil at the bottom of the experimental trays (**Supplementary Table 4**). Consequently, the EC at the top of the soil was more affected by the soil moisture level than the EC of the soil at the bottom in the combined stress treatment (**Supplementary Figure 2**).

**Figure 2:**
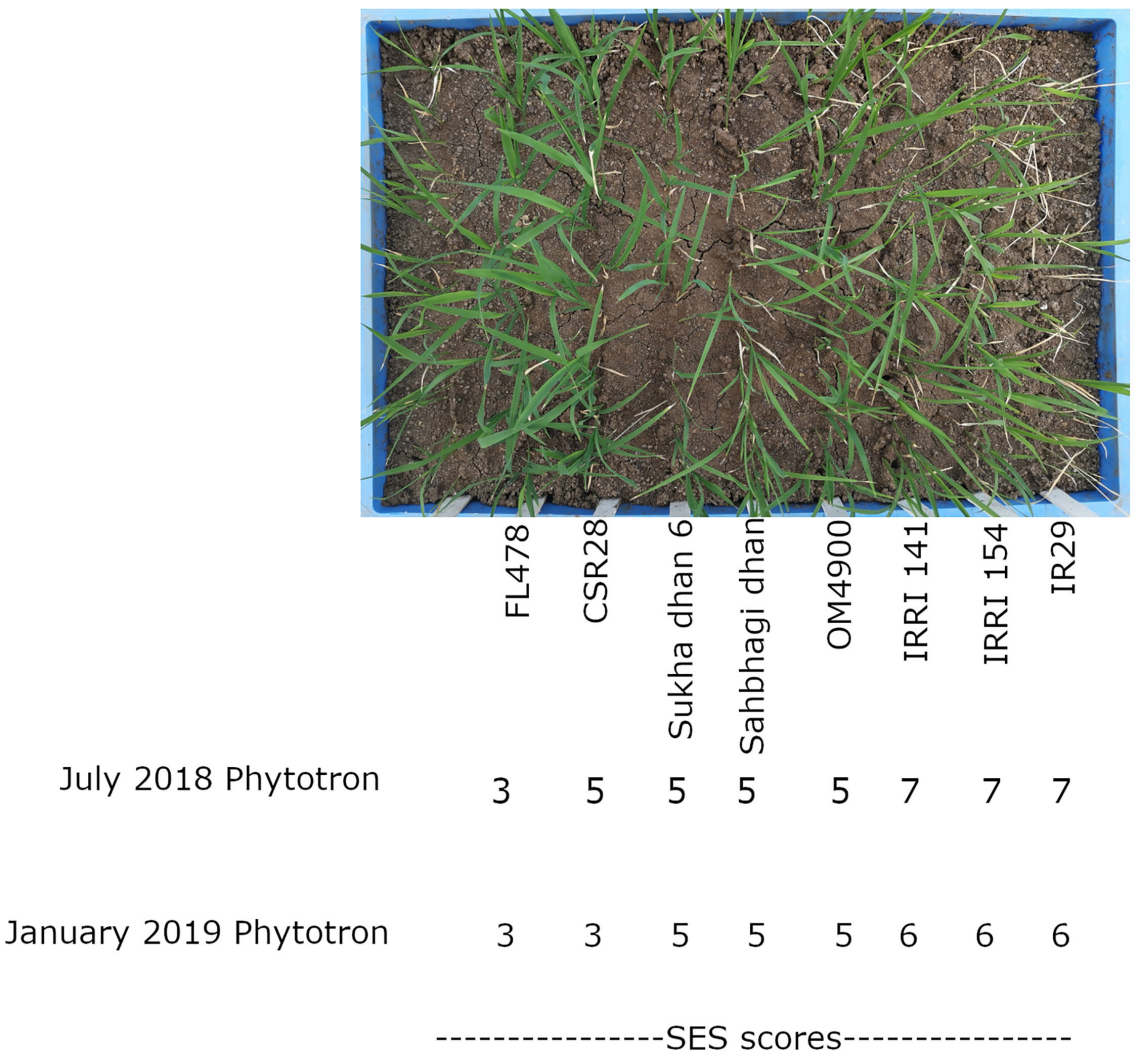
Visual scoring of the optimized drought+salinity treatment (T31; see **Supplementary Table 1**) and average SES scores across replicates for each variety in the July 2018 Phytotron and January 2019 Phytotron experiments.

**Figure 3:**
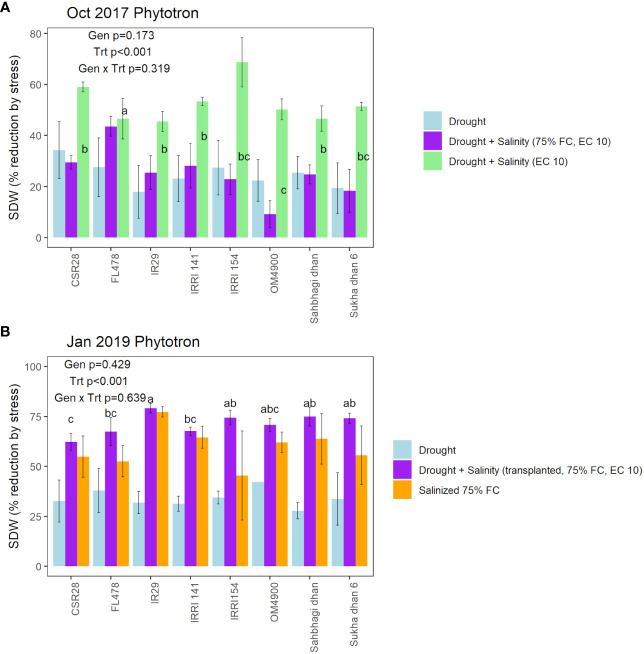
Genotypic and treatment effects on shoot dry weight reduction by stress in comparison with the control treatment in **(A)** the Oct 2017 Phytotron experiment (T5, T12, T13 in comparison with T16; see **Supplementary Table 1**), and **(B)** the January 2019 Phytotron experiment (T29, T30, T31 in comparison with T32, see **Supplementary Table 1**). Genotype, treatment, and genotype × treatment effects based on ANOVA are indicated in each panel. Significant differences among genotypes, which are indicated for the treatments in which the genotype effect was significant (p<0.05), are indicated by letter groups as determined by LSD test.

Although visual (SES) scoring is commonly used to identify genotypic differences in seedling stage salinity experiments, it is less commonly used in seedling stage drought experiments. We therefore considered several approaches for distinguishing among genotypes: 1) visual scoring, 2) destructive sampling (shoot dry weight), and physiological measurements (chlorophyll fluorescence, ion content, and chlorophyll concentration index, as well as stomatal conductance in the field experiment). Visual scoring was used as a deciding factor regarding the effectiveness of each treatment tested throughout the protocol optimization process (as listed in **Supplementary Table 1**). Using the optimized drought+salinity protocol, consistent differences in SES score were observed (**Figure 2**), with IR29, IRRI141, and IRRI154 exhibiting the highest SES scores and FL478 exhibiting the lowest scores. Treatment effects on SDW and SDW reduction by stress were significant in all physiology experiments (**Supplementary Table 5**; **Figure 3**; **Supplementary Figure 3**), but not in the field experiment as SDW was measured before the treatment differences were imposed. Genotype effects were significant (p<0.05) in the two drought+salinity treatments starting with soil moisture at 75% at field capacity (**Figure 3**).

**Figure 4:**
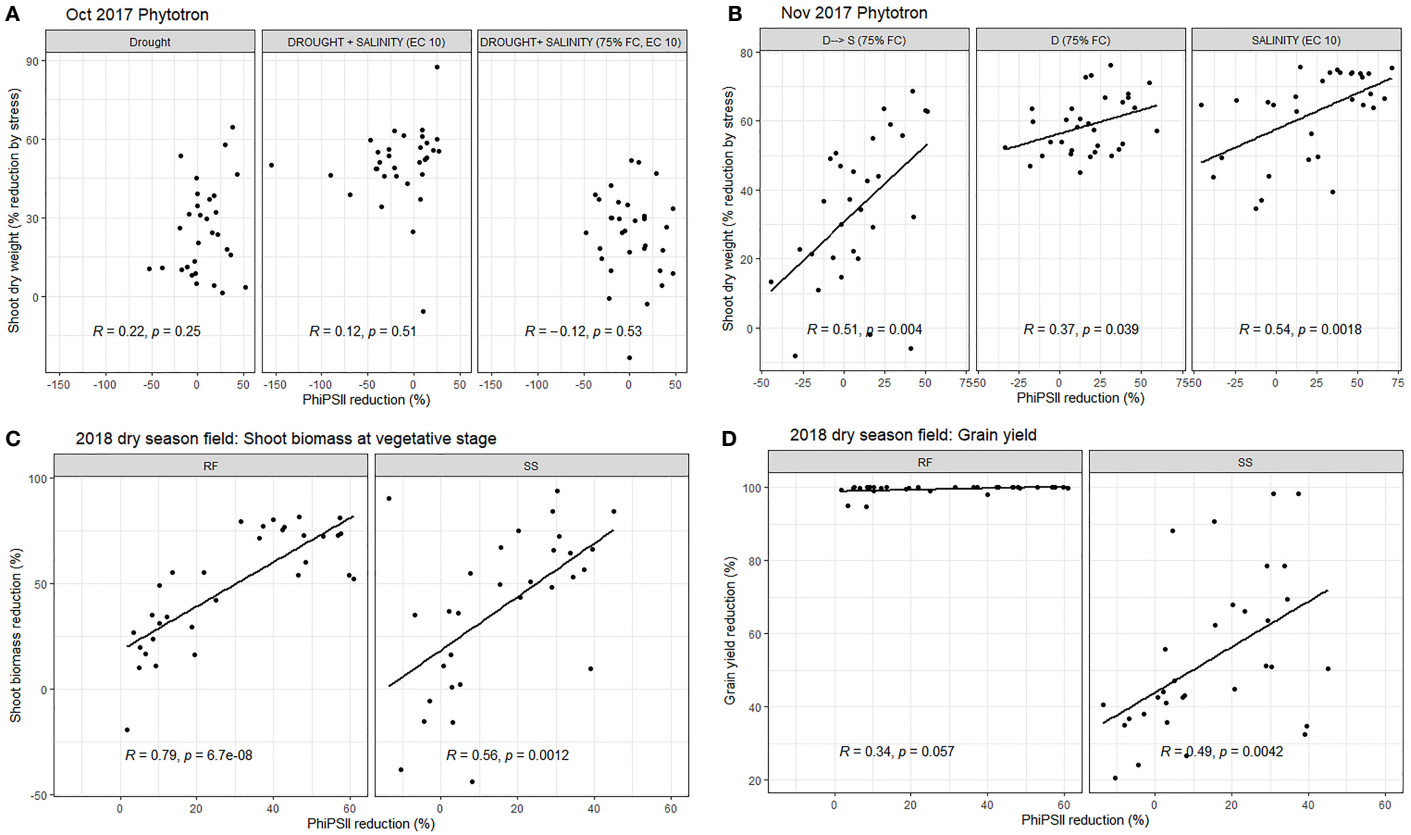
The relationship between reduction in chlorophyll fluorescence and shoot dry weight in the phytotron experiments in **(A)** October 2017 at 20 DAS (treatments T3, T12, T 13, and T16: see **Supplementary Table 1**), **(B)** November 2017 at 18 DAS (treatments T5, T14, T15, and T16: see **Supplementary Table 1**), and **(C)** in the 2018 dry season field trial at 45 DAS as well as **(D)** the relationship between reduction in chlorophyll fluorescence measured at 45 DAS and grain yield in the 2018 dry season field trial. All % reduction values were calculated as $\frac{x_{stress}-{\bar{x}}_{control}}{{\bar{x}}_{control}}\times 100$ RF, rainfed field treatment; SS, seedling/early vegetative stage field drought treatment.

On the replicated data, chlorophyll fluorescence (PhiPSII and PhiPSII reduction by stress) was most consistently negatively correlated with reduction in shoot dry weight in the stress treatments (**Supplementary Table 7**; **Figure 4**), as well as the SES score in the salinity treatments and Na^+^, K^+^, and Na^+^/K^+^ ratio in the salinity and drought+salinity treatments of the Jan 2019 Phytotron experiment (**Supplementary Table 7**). PhiPSII was significantly affected by treatment across experiments, except for the early measurement date in the field experiment (**Supplementary Table 6**). Chlorophyll concentration index, however, was not consistently significantly related with shoot dry weight reduction by stress across experiments (**Supplementary Table 7**). Stomatal conductance was not correlated with maintenance of SDW in the field drought experiment (**Supplementary Table 7**).

**Figure 5:**
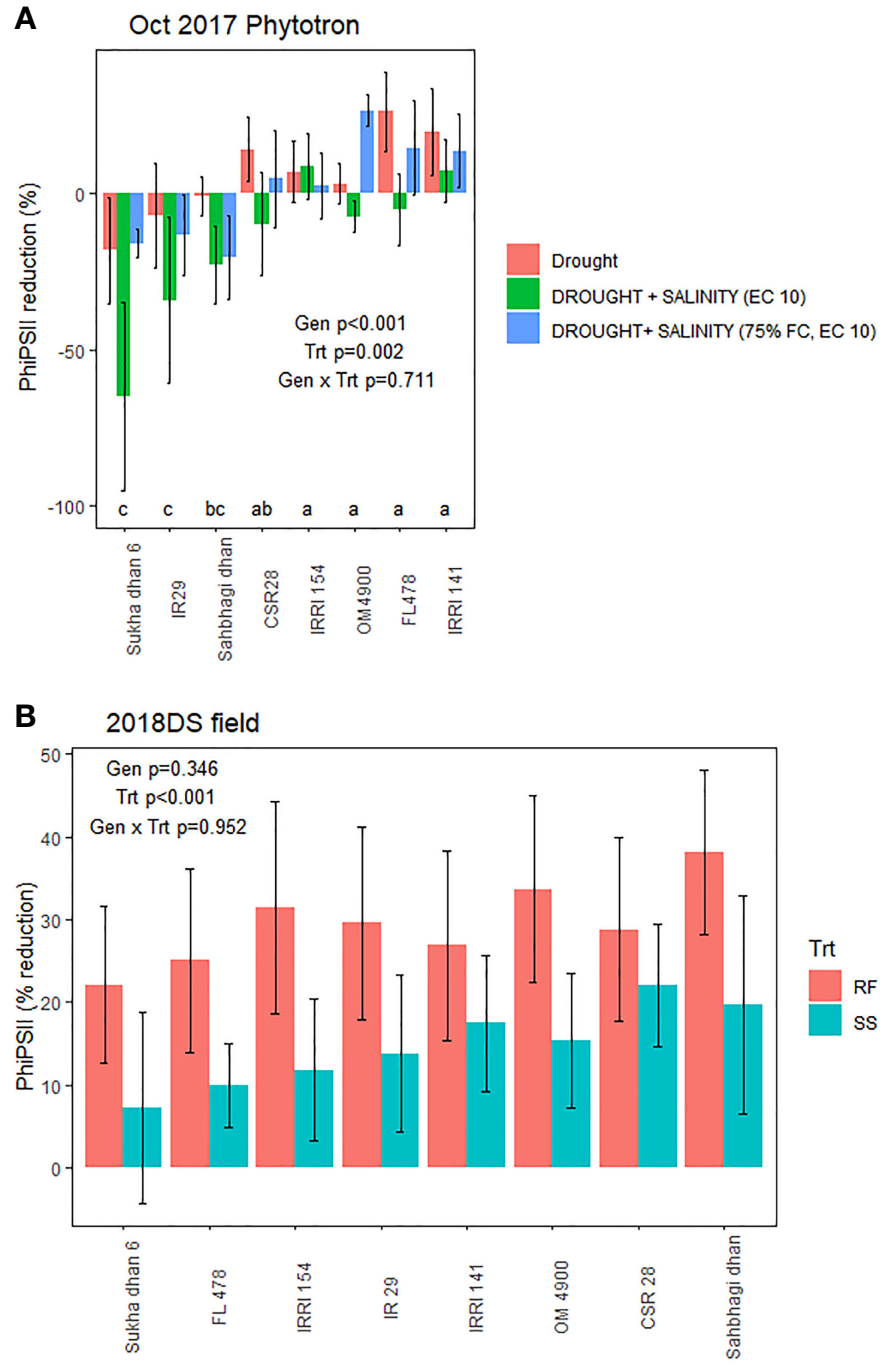
Treatment and genotypic effects chlorophyll fluorescence (PhiPSII) reduction by stress in **(A)** the October 2017 Phytotron experiment at 20 DAS (treatments T3, T12, T 13, and T16: see **Supplementary Table 1**) and **(B)** the 2018 dry season field trial at 45 DAS. Genotype, treatment, and genotype x treatment effects based on ANOVA are indicated.

Genotype effects on SDW indicated CSR28 and FL478 as having larger values across treatments in multiple experiments (**Supplementary Table 5**), but most experiments showed no significant differences in the absolute PsiPSII values among genotypes (**Supplementary Table 6**). Genotype effects on reduction of PhiPSII were significant across treatments in the Oct 2017 Phytotron experiment, but not in the Nov 2017 Phytotron or the field experiment (**Figure 4**; **Supplementary Figure 4**). Among genotypes, Sukha dhan 6 showed trends of least reduction in PhiPSII across treatments in the Oct 2017 Phytotron experiment (p<0.001) and also showed least (although non-significant) reduction in PhiPSII in the field experiment (**Figure 5**).

## 4 Discussion

Abiotic stresses are highly complex, and more than one stress can simultaneously affect crop growth in farmers’ fields. Screening and identification of tolerant cultivars requires a reliable phenotyping methodology. Most abiotic stress screening studies have focused on selection under individual stresses or screening for one stress followed by the screening for a second or third stress in the methodology of development of multiple stress tolerant cultivars (Gregorio et al., 2013). In combined screening for more than one stress, types of stress that require similar screening conditions are more straightforward to execute than the stresses that require very different screening conditions. For example, combined salinity and submergence screening involves salinizing the water used for submergence; this is a simpler protocol to develop than screening for salinity and drought – one of which requires the presence of water and the other of which requires the absence. By testing a range of individual stress intensities and timings of applying the individual stresses in respect to each other, we were able to develop a seedling stage drought+salinity screening protocol in which both stresses were initiated together (i.e. adding salinized water at deficit levels), which resulted in clearly visible plant stress symptoms.

The cultivar differences under our optimized seedling stage drought+salinity screening protocol were distinct from those in the individual drought and salinity treatments (**Figure 4**; **Supplementary Figure 3**), confirming the combined stress treatment as a distinct type of stress compared to the individual stresses. Recent research in barley and Medicago reported that plant response to combined abiotic stress is unique and different to that of response to individual stresses including drought, ozone, and heat (Iyer et al., 2013; Rollins et al., 2013). Mittler (2006) also concluded that plant response to a combination of two different abiotic stresses is ‘unique’ and cannot be directly extrapolated from the response to each of the different stresses applied individually, as experienced during our efforts to develop the protocol for sequential verses simultaneous stresses. Therefore, it is imperative to develop a robust phenotyping protocol for combined drought+salinity to breed rice varieties specifically for this unique stress.

The level of salinity stress (EC) at the top soil layer of our drought+salinity screening system was relatively higher than that at the bottom soil layer (**Supplementary Table 4**), which is likely due to differences in the moisture content and reduced percolation of salts to the bottom soil layers. This stratification likely explains why the plant response to treatment of drought followed by salinity was different from that under salinity followed by drought, in which the plants performed much better when drought was applied first; the drought stress likely induced deeper root growth, which subsequently allowed the plants to avoid the higher salt concentrations in the top layer of the soil. These conditions of the combined drought+salinity screening protocol developed in this study apply to field conditions in which the crop initially experiences drought due to deficit in rainfall and where there is a risk of salinity, such as in coastal zones. The stratification of salt in the drought+salinity treatment here, which likely favors deeper root growth, is in contrast to recently reported results of surface rooting as beneficial under (non-drought stressed) saline conditions (Kitomi et al., 2020). A better understanding of the stratification of salt along the soil profile in target environments is necessary.

We conducted a range of measurements to understand how the plants were responding to each treatment as the seedling stage drought+salinity protocol was being optimized. Although SES scores (reflecting salt-induced senescence) are typically employed for salinity screening, and growth parameters such as maintenance of SDW (reflecting the ability to access water in drying soil) are typically employed for vegetative stage drought stress screening, functional parameters such as chlorophyll fluorescence may be an ideal approach to integrate both types of responses that are relevant to a drought+salinity treatment. It was also notable that chlorophyll fluorescence correlated with grain yield when both the stress applied and the chlorophyll measurements were conducted during the vegetative stage only, and not when conducted later in the growth cycle (**Figure 4**), highlighting the effectiveness of targeting applied stresses to specific growth stages (e.g. Fukai et al., 1999; Zeng et al., 2001). The inconsistent correlations of CCI with SDW reduction by stress may be due to the opposing effects of drought and salinity on rice leaf chlorophyll concentration, wherein salinity generally decreases chlorophyll concentration as related to senescence (Lutts et al., 1996) and drought may increase chlorophyll concentration depending on the type of drought stress (Barnaby et al., 2019).

Since the method optimized here could visibly distinguish the performance the cultivars as evidenced by the SES scores (**Figure 2**), this optimized protocol can be used for screening in a breeding program to improve popular but stress-susceptible cultivars that are grown in regions prone to seedling stage combined drought+salinity stress. Further optimization is required to apply this protocol to the development of combined drought+salinity treatment as a field phenotyping method, to develop varieties that can perform well in the areas such as the Mekong River Delta of Vietnam where both stresses coincide.

## 5 Conclusions

In this study, we optimized a screening protocol for seedling stage combined drought+salinity stress in rice, in which both stresses are applied simultaneously by transplanting seedlings into saline soil at 75% of field capacity which is then allowed to undergo progressive drydown. Based on genotypic differences and correlations among physiological traits, the combined stress treatment appeared to have effects on the rice plants that are distinct from those of individual drought and salinity stress. Although visual scoring may be an effective screening criteria, our results suggested chlorophyll fluorescence conducted during stage-specific stress treatment as an effective and potentially high-throughput approach to screen genotypes for their response to combined drought+salinity at seedling stage. Future work characterizing drought+salinity -prone environments can help further define regions where this protocol can be targeted and used in the development of new rice varieties tolerant to combined stresses.

## Data availability statement

The original contributions presented in the study are included in the article/**Supplementary Material**. Further inquiries can be directed to the corresponding authors.

## Author contributions

RS, AH, AK, FM and SK designed this experiment. SK, NV, CLUC, and CAC executed the experiments and collected the data. MQ, JE and AH were responsible for the statistical analysis. SK and AH completed the writing of the original manuscript. RS, AH, AK, FM and SK were responsible for the manuscript verification. All authors contributed to the article and approved the submitted version.
