# Supplementary material for: Development of a phenotyping protocol for combined drought and salinity stress at seedling stage in rice
Source: Front Plant Sci. 2023 May 31;14:1173012. doi: 10.3389/fpls.2023.1173012 (PMC10266585; doi:10.3389/fpls.2023.1173012)
Supplement: Supplementary file 1 [file DataSheet_1.pdf]

Supplementary Material

1 Supplementary Figures and Tables

1.1 Supplementary Tables

Supp. Table 1: Details of treatments tested in different experiments of optimization of salinity and drought phenotyping. FC: field capacity, EC: electrical conductivity, MC: moisture content, DAS: days after seeding. After the initial addition of saline water, soil moisture levels were maintained with tap water.

A. Testing different levels of individual salinity stress

Treatment No.	Treatment details
T1	Seeding of pre-germinated seeds at FC (field capacity), salinized EC4 dSm^{-1}after seedling emergence and establishment, maintained at FC
T2	Pre-germinated seeds seeding at FC, salinized EC8 dSm^{-1}after seedling emergence and establishment, maintained at FC
T3	Pre-germinated seeds seeding at FC, salinized EC10 dSm^{-1}after seedling emergence and establishment, maintained at FC
T4	Pre-germinated seeds seeding at FC, salinized EC12 dSm^{-1}after seedling emergence and establishment, maintained at FC

B. Testing different orders of applying salinity and drought stress, with different levels of salinity but all soil moisture levels starting at field capacity

T5	Pre-germinated seeds seeding at FC, drought imposition after seedling emergence and establishment by no application of water for 15 days
T6	Pre-germinated seeds seeding at FC, drought first (for 15 days) then salinity (EC8 dSm^{-1}at FC) at30% of FC (for another 15 days)
T7	Pre-germinated seeds seeding at FC, drought first (no application of water for15 days) then salinized (EC 8 dSm^{-1}) at FC (for another15 days at FC)
T8	Pre-germinated seeds seeding at FC, salinity first (EC4 dSm^{-1} at FC for 15 days) after seedling emergence and establishment then drought (for another 15 days)
T9	Pre-germinated seeds seeding at FC, salinization at seeding (EC4 dSm^{-1} at FC) followed by drought (15 days)
T10	Pre-germinated seeds seeding at FC, salinization (EC8 dSm^{-1} at FC) after 4 DAS followed by drought (15 days)
T11	Pre-germinated seeds seeding at FC, drought first (15 days) then salinity (EC 8 dSm^{-1}) at 25% of FC (15 days)
T12	Pre-germinated seeds seeding at FC, salinization (EC10 dSm^{-1}at FC) at seeding followed by no water application

C. Testing treatments initiated at soil moisture levels below field capacity

T13	Seeding at 75% of FC, salinization (EC10 dSm^{-1}at FC) at seeding followed by no water application
T14	Seeding at 75% of FC followed by drought (no water application)
T15	Seeding at 75% of FC, MC reduced to 30% of FC (moderate drought) then salinity (EC 10 dSm^{-1}at FC) for 15 days
T16	Control treatment: seeding at FC & maintained at FC

D. Testing treatments allowed to dry down to 30% of field capacity only

T17	Seeding at FC, salinized (EC 10 dSm^{-1}at FC) after seedling emergence and establishment, MC reduced to 30% of FC, further MC maintained at 30% FC
T18	Seeding at FC followed drought by no application of water until 30% of FC, further maintained at 30% of FC
T19	Seeding at FC followed drought until the end of experiment by no application of water
T20	Seeding at FC, moisture reduced to 30 % of FC (moderate drought), salinized at 30% FC with EC10 dSm^{-1}at FC), maintained at 30% FC

E. Monitoring soil moisture evaporation in unplanted trays across different treatments

T21	Trays with soil at 100% FC (normal water) + no seeding
T22	Trays with soil salinized at EC 10 dSm^{-1}, maintained at 100% FC + no seeding
T23	Trays with soil at 100% FC, moisture reduced to 30% FC; maintained at 30% FC (drought at 30% FC)+ no seeding
T24	Trays with soil at 100% FC at beginning, no water added until end of the experiment + no seeding
T25	Trays with soil at 100% FC, moisture reduced to 30% of FC; at 30% FC salinized EC10 dSm^{-1}; maintained at 30% FC+ no seeding

F. Comparing direct seeding with transplanting young seedlings

T26	Soil salinized with EC10 dSm^{-1} at FC; MC of trays reduced to 60%FC, direct seeding of dry seeds at 60%FC of previously salinized trays
T27	Soil salinized with EC10 dSm^{-1} at FC; MC of trays reduced to 60%FC, transplanted young seedlings (7 days old) at 60% FC of previously salinized trays
T28	Unplanted trays with salinized soil at EC10 dSm^{-1} to monitor moisture evaporation

G. Finalizing the optimized seedling stage drought+ salinity protocol

T29	Transplanted 7 day old seedlings in soil salinized at MC of 75% FC (EC10 dSm^{-1}at FC), further moisture level maintained at 75% of FC
T30	Transplanted 7 day old seedlings in soil at 75% FC moisture and allowed to experience drought until 30% of FC, maintained at 30% FC
T31	Transplanted 7 day old seedlings in soil salinized at MC of 75% FC (EC10 dSm^{-1} at FC), further moisture level reduced to 30% FC by drying, and maintained at 30 % of FC
T32	Transplanted 7 day old seedlings in soil at 75% FC moisture and maintained at 75% FC as control

Supp Table 2: SNAP Solution used for salinity screening of the backcross generations. SNAP A and B stocks were provided by the University of the Philippines Los Baños Institute of Plant Breeding.

Stock		Liters
1	SNAP A	1% of total volume of water
2	SNAP B	1% of total volume of water
3	$FeSO_4$ (made fresh)	2.5 g/L; 1.25 ml of the solution per liter of water

Supp Table 3. Standard Evaluation Scores for seedling stage salinity screening through solution culture method (as described in IRRI, 1996).

SES	Observation	Tolerance
1	Normal growth, only the old leaves show white tips while no symptoms on young leaves	Very high
3	Near normal growth, but only leaf tips burn, few older leaves become whitish partially	High
5	Growth severely retarded; most old leaves severely injured, few young leaves elongating	Moderate
7	Complete cessation of growth; most leaves dried; only few young leaves still green	Susceptible
9	Almost all plants dead or dying	Highly Susceptible

Supp. Table 4. Soil electrical conductivity (EC) levels in the Phytotron physiology, drought+salinity standardization (shown in Fig. 1), and intercross screening experiments. Values shown are the EC of the soil extract (ECe) in dS m^{-1}.

Expt	Treatment No. (Supp. Table 3)	Treatment	Bulk soil	Top	Middle	Bottom
Oct 2017 Phytotron	T16	CONTROL	4.7			
	T5	Drought	7.1			
	T12	DROUGHT + SALINITY (EC 10)	15.9			
	T13	DROUGHT+ SALINITY (75% FC; EC 10)	7.8			
Nov 2017 Phytotron	T16	CONTROL	3.7			
	T14	D (75% FC)	4.0			
	T15	D--> S (75% FC)	6.5			
	T3	SALINITY (EC 10)	4.4			
July 2018: D+S standardization	T32	Control		3.6	4.0	3.8
	T30	Drought		4.0	4.2	3.8
	T31	DROUGHT+ SALINITY (75% FC; EC 10)		17.6	14.4	14.9
	T29	Salinized 75% FC		12.8	10.1	12.4
Dec 2018: D+S standardization	T31	DROUGHT+ SALINITY (75% FC; EC 10)		12.4		9.7
Oct 2020: D+S standardization	T31	DROUGHT+ SALINITY (75% FC; EC 10)		15.7	7.1	6.7

Supp. Table 5. Absolute values of shoot dry weight at seedling stage across treatments in the October 2017 Phytotron, November 2017 Phytotron, 2018 Field, and January 2019 Phytotron experiments. P values from ANOVA across treatments and for the genotype effect within each treatment are shown. Values shown are shoot dry weight in grams per plant for the phytotron experiments and grams per 2 hills for the field experiment.

**October 2017 Phytotron**								
Treatment	<0.001							
Genotype	<0.001							
Trt x Gen	0.541							
	Control		Drought		DROUGHT + SALINITY (EC 10)		DROUGHT+ SALINITY (75% FC, EC 10)	
CSR28	0.138	a	0.091		0.057	ab	0.097	a
FL478	0.138	a	0.100		0.074	a	0.078	b
IR29	0.074	b	0.061		0.050	b	0.064	bc
IRRI 141	0.089	b	0.068		0.041	b	0.064	bc
IRRI 154	0.095	b	0.069		0.037	b	0.073	bc
OM4900	0.088	b	0.068		0.044	b	0.080	ab
Sahbhagi dhan	0.079	b	0.059		0.042	b	0.059	c
Sukha dhan 6	0.085	b	0.069		0.042	b	0.070	bc
Gen	0.01		0.116		0.0158		0.007	

**November 2017 Phytotron**						
Treatment	<0.001					
Genotype	<0.001					
Trt x Gen	0.223					
	CONTROL		D (75% FC)		D--> S (75% FC)	SALINITY (EC 10)
CSR28	0.169	a	0.058	ab	0.076	0.061
FL478	0.157	a	0.064	a	0.114	0.059
IR29	0.100	b	0.049	bc	0.073	0.034
IRRI 141	0.098	b	0.043	c	0.059	0.039
IRRI 154	0.088	b	0.036	c	0.053	0.032
OM4900	0.103	b	0.045	bc	0.077	0.048
Sahbhagi dhan	0.099	b	0.039	c	0.069	0.034
Sukha dhan 6	0.092	b	0.036	c	0.067	0.032
Gen	0.002		0.004		0.078	0.130

Field 2018DS			
Treatment	<0.001		
Genotype	0.85		
Trt x Gen	0.887		
	WW	SS	RF
CSR 28	13.11	6.07	6.24
FL 478	12.78	10.26	7.78
IR 29	11.94	5.63	6.22
IRRI 141	13.05	6.91	5.36
IRRI 154	10.19	9.94	6.55
OM 4900	12.72	9.55	5.24
Sahbhagi Dhan	10.64	10.16	5.74
Sukha dhan 6	15.77	8.03	7.11
Gen	0.41	0.85	0.97

January 2019 Phytotron						
Treatment	<0.001					
Genotype	<0.001					
Trt x Gen	0.956					
	CONTROL		DROUGHT	Drought + Salinity		SALINIZED 75% FC
CSR28	0.182	a	0.123	0.069	a	0.082
FL478	0.173	ab	0.107	0.056	ab	0.082
IR29	0.110	d	0.075	0.023	d	0.025
IRRI 141	0.134	bcd	0.092	0.043	bc	0.048
IRRI154	0.153	abc	0.100	0.039	bcd	0.083
OM4900	0.112	cd	0.087	0.033	cd	0.043
Sahbhagi Dhan	0.133	bcd	0.096	0.033	cd	0.048
Sukha dhan 6	0.129	cd	0.085	0.033	cd	0.057
Gen	0.012		0.268	0.003		0.090

Supp. Table 6. Absolute values of the quantum yield of photosystem II (PhiPSII: $[F_m' - F_t]/F_m'$), across treatments in the October 2017 Phytotron, November 2017 Phytotron, and 2018 Field experiments. P values from ANOVA across treatments and for the genotype effect within each treatment are shown. Values shown are shoot dry weight in grams per plant for the phytotron experiments and grams per 2 hills for the field experiment.

**October 2017 Phytotron**				
Treatment	0.0472			
Genotype	0.0208			
Trt x Gen	0.9616			
	CONTROL	Drought	DROUGHT + SALINITY (EC 10)	DROUGHT+ SALINITY (75% FC, EC 10)
CSR28	0.175	0.150	0.192	0.167
FL478	0.171	0.126	0.180	0.146
IR29	0.129	0.139	0.174	0.147
IRRI 141	0.150	0.121	0.140	0.130
IRRI 154	0.154	0.143	0.141	0.150
OM4900	0.141	0.137	0.151	0.104
Sahbhagi dhan	0.107	0.109	0.132	0.129
Sukha dhan 6	0.101	0.119	0.166	0.117
Gen	0.411	0.662	0.506	0.295

**November 2017 Phytotron**				
Treatment	0.00515			
Genotype	0.426			
Trt x Gen	0.998			
	CONTROL	D (75% FC)	D--> S (75% FC)	SALINITY (EC 10)
CSR28	0.357	0.293	0.313	0.277
FL478	0.344	0.264	0.359	0.288
IR29	0.362	0.275	0.314	0.219
IRRI 141	0.304	0.256	0.291	0.259
IRRI 154	0.318	0.300	0.227	0.242
OM4900	0.367	0.290	0.371	0.300
Sahbhagi dhan	0.295	0.247	0.293	0.223
Sukha dhan 6	0.332	0.283	0.267	0.237
Gen	0.730	0.979	0.373	0.964

**Field 2018DS 45 DAS**			
Treatment	<0.001		
Genotype	0.998		
Trt x Gen	0.999		
	WW	SS	RF
CSR 28	0.449	0.350	0.320
FL 478	0.436	0.393	0.327
IR 29	0.441	0.380	0.311
IRRI 141	0.436	0.360	0.319
IRRI 154	0.447	0.394	0.306
OM 4900	0.452	0.382	0.300
Sahbhagi dhan	0.455	0.365	0.281
Sukha dhan 6	0.405	0.375	0.315
Gen	0.456	0.992	0.999

**Field 2018DS 111 DAS**					
Treatment	<0.001				
Genotype	<0.001				
Trt x Gen	0.013797				
	WW	SS		RF	
CSR 28	0.301	0.327	ab	0.330	ab
FL 478	0.289	0.186	cd	0.297	abc
IR 29	0.256	0.368	a	0.292	bc
IRRI 141	0.211	0.236	bc	0.286	bc
IRRI 154	0.325	0.254	bc	0.358	ab
OM 4900	0.296	0.231	bc	0.382	a
Sahbhagi dhan	0.279	0.128	d	0.276	bc
Sukha dhan 6	0.249	0.193	cd	0.228	c
Gen	0.396	0.001		0.040	

Supp. Table 7. Correlations among physiological traits measured in the Phytotron experiments in A) October 2017 at 20 DAS, B) November 2017 at 18 DAS, C) July 2018 at 11 DAT, and C) in the 2018 dry season field trial at 45 and 101 DAS. *: p<0.05, **: p<0.01, ***:p<0.001. Treatments shown (as indicated by their treatment number) are described in Supp. Table 1.

PhiPSII: efficiency of Photosystem II, CCI: chlorophyll concentration index, SDW: shoot dry weight, gs: stomatal conductance, CT: canopy temperature, HI: harvest index, DTF: days to 50% flowering, GY: grain yield, % red: percent reduction in the stress treatment compared to the well-watered control treatment, DAS: days after sowing.

All % reduction values were calculated as $\frac{\bar{x}_{control} - x_{stress}}{\bar{x}_{control}} \times 100$, and % increase values were calculated as $\frac{x_{stress} - \bar{x}_{control}}{\bar{x}_{control}} \times 100$.

A) Phytotron Oct 2017

	CCI	SDW	PhiPSII %red	CCI% red	SDW% red
**Drought (T5)**					
PhiPSII	0.5	0.17	-0.69***	-0.44*	-0.06
CCI		0.17	-0.26	-0.95***	-0.02
SDW			0.15	-0.09	-0.62***
PhiPSII % red				0.23	0.22
CCI % red					0.04
**DROUGHT + SALINITY (EC 10) (T12)**					
PhiPSII	0.26	0.22	-0.76***	-0.22	-0.01
CCI		0.23	-0.17	-0.96***	-0.23
SDW			0.05	-0.22	-0.67***
PhiPSII % red				0.1	0.12
CCI % red					0.27
**DROUGHT+ SALINITY (75% FC, EC 10) (T13)**					
PhiPSII	-0.15	-0.06	-0.71***	0.19	0.37*
CCI		0.07	0.11	-0.92***	-0.2
SDW			0.4*	0.03	-0.43*
PhiPSII % red				-0.21	-0.12
CCI % red					0.17

B) Phytotron Nov 2017

	CCI	SDW	PhiPSII %red	CCI% red	SDW% red	SES
**SALINITY (EC 10) (T3)**						
PhiPSII	0.19	0.53**	-0.98***	-0.24	-0.56***	-0.4*
CCI		-0.14	-0.22	-0.89***	0.02	0.22
SDW			-0.46**	0	-0.8***	-0.62***
PhiPSII % red				0.21	0.54**	0.33
CCI % red					0.04	-0.08
SDW % red						0.33
**D--> S (75% FC) (T15)**						
PhiPSII	0.27	0.64***	-0.96***	-0.3	-0.48**	-0.27
CCI		0.46*	-0.25	-0.94***	-0.17	-0.22
SDW			-0.59***	-0.44*	-0.75***	-0.42*
PhiPSII % red				0.22	0.51**	0.24
CCI % red					0.06	0.29
SDW % red						0.16
**D (75% FC) (T14)**						
PhiPSII	0.12	0.25	-0.96***	-0.19	-0.37*	0.13
CCI		0.01	-0.18	-0.82***	-0.04	0.03
SDW			-0.14	-0.2	-0.5**	-0.08
PhiPSII % red				0.12	0.37*	-0.17
CCI % red					-0.04	-0.09
SDW % red						-0.13

C) Phytotron July 2018

	SDW	Na^+	K^+	$Na^+:K^+$	SES	SDW %red	Na^+ %inc	K^+ % inc
**DROUGHT (T30)**								
Na^+	-0.02							
K^+	-0.19	-0.32						
$Na^+:K^+$	-0.01	0.97***	-0.53*					
SES	-	-	-	-				
SDW %red	-0.56*	-0.05	0.42	-0.12	-			
Na^+ %inc	0	0.92***	-0.04	0.82***	-	-0.06		
K^+ % inc	-0.58*	-0.09	0.77***	-0.25	-	0.44	0.14	
$Na^+:K^+$ %inc	0.23	0.93***	-0.31	0.89***	-	-0.2	0.93***	-0.21
**SALINIZED 75% FC (T29)**								
Na^+	-0.58*							
K^+	0.31	-0.76***						
$Na^+:K^+$	-0.52*	0.99***	-0.79***					
SES	-0.46	0.54*	-0.58*	0.52*				
SDW %red	-0.94***	0.52*	-0.19	0.47*	0.3			
Na^+ %inc	-0.58*	0.98***	-0.77***	0.96***	0.53*	0.51*		
K^+ % inc	0.09	-0.57*	0.89***	-0.62**	-0.31	-0.01	-0.58*	
$Na^+:K^+$ %inc	-0.51*	0.98***	-0.82***	0.98***	0.5*	0.44	0.98***	-0.68**
**DROUGHT+ SALINITY (75% FC, EC 10) (T31)**								
Na^+	-0.38							
K^+	0.33	-0.72***						
$Na^+:K^+$	-0.5*	0.9***	-0.89***					
SES	-0.79***	0.25	-0.02	0.31				
SDW %red	-0.92***	0.43	-0.48*	0.61**	0.64**			
Na^+ %inc	-0.27	0.89***	-0.47*	0.69**	0.22	0.27		
K^+ % inc	0.03	-0.64**	0.92***	-0.76***	0.29	-0.19	-0.42	
$Na^+:K^+$ %inc	-0.36	0.94***	-0.78***	0.9***	0.16	0.44	0.9***	-0.71***

D) 2018 dry season field, seedling/early vegetative stage drought stress

	SDW 45 DAS	GY
SDW 45 DAS		0.51**
PhiPSII 45 DAS	0.63***	0.4*
PhiPSII 111 DAS	-0.38*	-0.44*
CCI 45 DAS	0.64***	0.25
CCI 111 DAS	0.36*	0.26
gs 111 DAS	0.41*	0.26
CT 111 DAS	0.16	-0.18
Biomass at harvest	0.36*	0.58***
HI	0.42*	0.91***
DTF	-0.27	-0.62***
GY % red	-0.54**	-0.91***
SDW 45 DAS % red	-0.96***	-0.62***
PhiPSII 45 DAS % red	-0.6***	-0.44*
PhiPSII 111 DAS % red	0.41*	0.44*
CCI 45 DAS % red	-0.61***	-0.36*
CCI 111 DAS % red	-0.51**	-0.46**
gs 111 DAS % red	-0.32	-0.09

1.2 Supplementary Figures

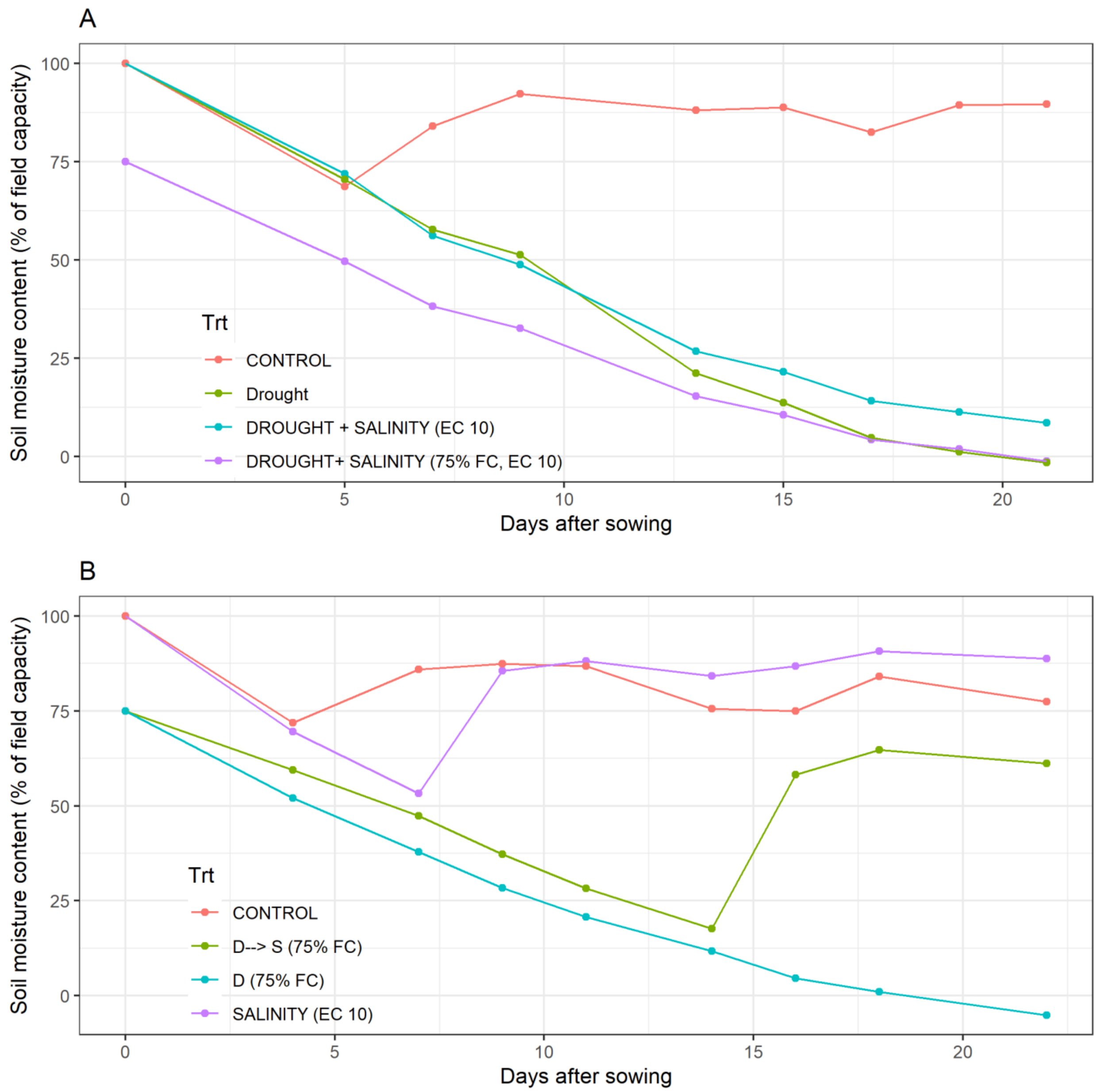

Supplementary Figure 1. Soil moisture levels in the Phytotron physiology experiments as a % of field capacity. A) the October 2017 experiment (T5, T12, T13, T16; see Supp. Table 1), and B) the November 2017 experiment (T3, T14, T15, T16; see Supp. Table 1).

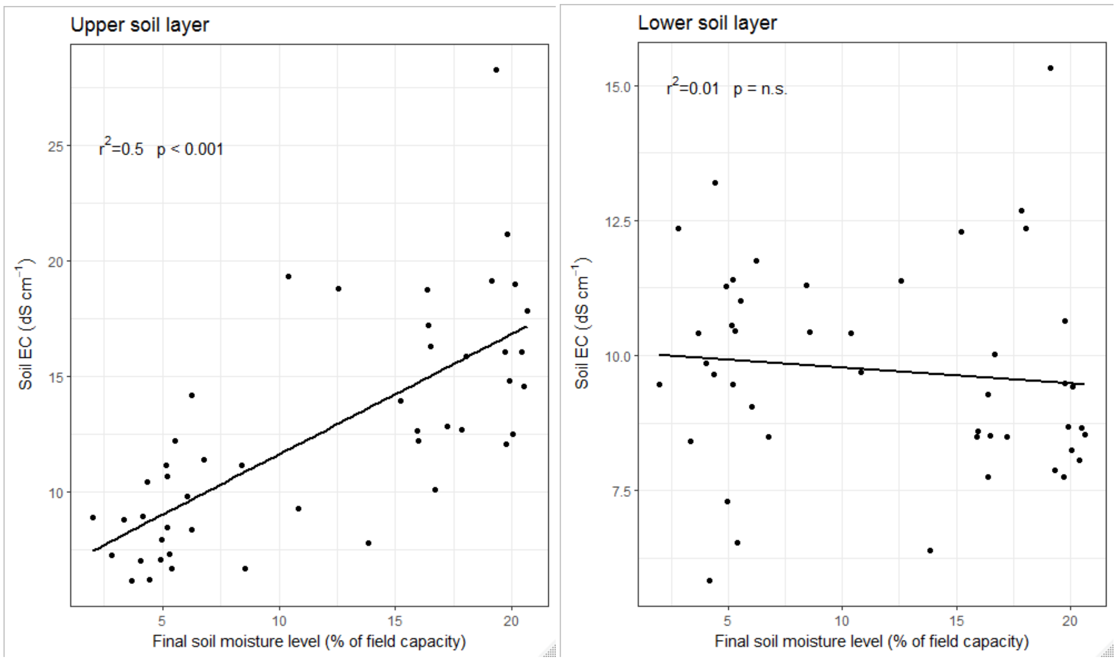

Supplementary Figure 2. Relationship between soil moisture levels and soil EC values at the end of the combined drought+ salinity stress (T31).

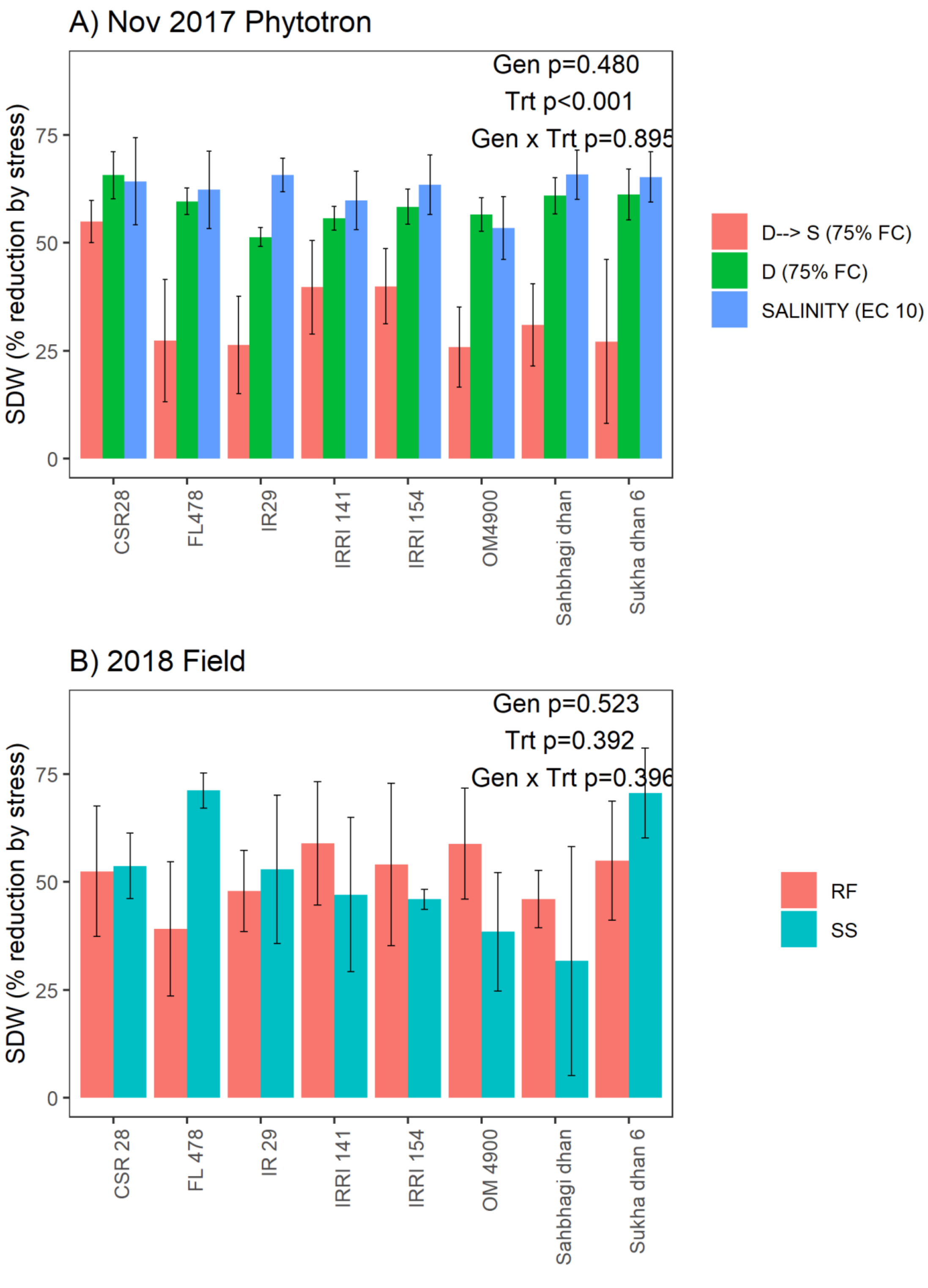

Supplementary Figure 3. Treatment and genotypic effects shoot dry weight reduction by stress in the A) Nov 2017 Phytotron (T3, T14, T15, T16; see Supp. Table 1), and B) 2018 dry season field experiments (RF: rainfed treatment, SS: drought treatment at seedling stage only). Genotype, treatment, and genotype x treatment effects based on ANOVA are indicated in each panel.

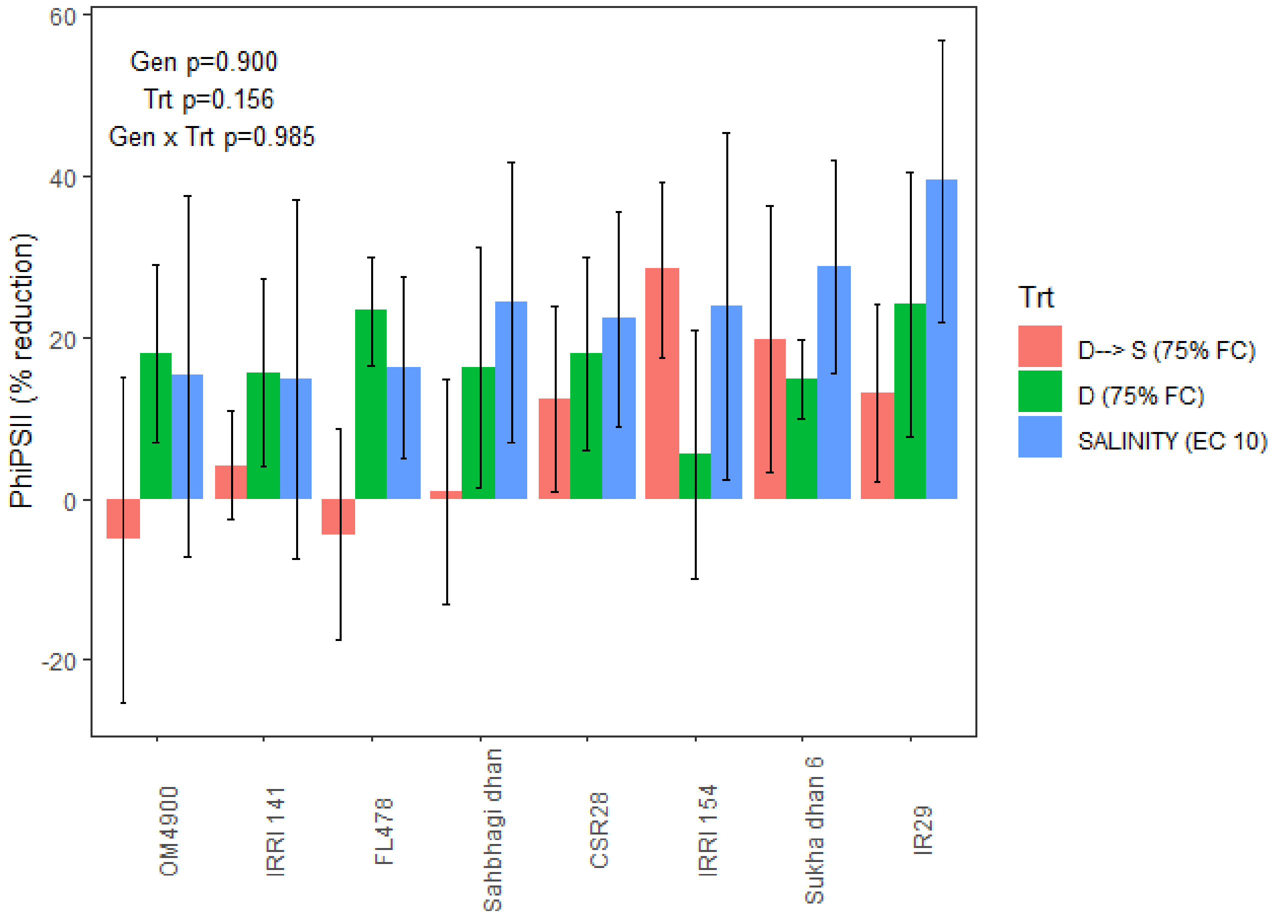

Supplementary Figure 4. Treatment and genotypic effects chlorophyll fluorescence (PhiPSII) reduction by stress in the Nov 2017 Phytotron (T3, T14, T15, T16; see Supp. Table 1) experiment. Genotype, treatment, and genotype x treatment effects based on ANOVA are indicated.
